# Resident Perceptions of a Publicly Disclosed Daily Productivity Dashboard

**DOI:** 10.5811/westjem.2021.10.53874

**Published:** 2022-01-03

**Authors:** Katja Goldflam, Alina Tsyrulnik, Colin Flood, Jessica Bod, Ryan F. Coughlin, David Della-Giustina

**Affiliations:** Yale School of Medicine, Department of Emergency Medicine, New Haven, Connecticut

## Abstract

**Introduction:**

Following resident requests, we created a public metrics dashboard to inform residents of their daily productivity. Our goal was to iteratively improve the dashboard based on resident feedback and to measure the impact of reviewing aggregate data on self-perceived productivity.

**Methods:**

A 10-question anonymous survey was completed by our postgraduate year 1–3 residents. Residents answered questions on the dashboard and rated their own productivity before and after reviewing aggregate peer-comparison data. Using the Wilcoxon signed-rank test we calculated summary statistics for survey questions and compared distributions of pre- and post-test, self-rated productivity scores.

**Results:**

All 43 eligible residents completed the survey (response rate 100%). Thirteen (30%) residents “rarely” or “never” reviewed the dashboard. No respondents felt the dashboard measured their productivity or quality of care “extremely accurately” or “very accurately.” Seven (16%) residents felt “very” or “extremely pressured” to change their practice patterns based on the metrics provided, and 28 (65%) would have preferred private over public feedback. Fifteen residents (35%) changed their self-perceived rank after viewing peer-comparison data, although not significantly in a particular direction (z = 0.71, P = 0.48).

**Conclusion:**

Residents did not view the presented metrics as reflective of their productivity or quality of care. Viewing the dashboard did not lead to statistically significant changes in resident self-perception of productivity. This finding highlights the need for expanding the resident conversation and education on metrics, given their frequent inclusion in attending physician workforce payment and incentive models.

## INTRODUCTION

The number of patients seen per hour is a common metric used in evaluating the on-shift performance and productivity of attending emergency physicians.^1^–^4^ Sharing productivity metrics can have some impact on clinician performance^5^ and has been shown to increase emergency medicine (EM) resident satisfaction with their evaluation and feedback processes.^6^ The productivity of EM residents at various stages in training has been previously characterized,^7^–^9^ but there is little standardization in how residency programs use productivity data in resident education, what format is most useful, and how residents perceive and apply the data.^10^

Residents at our urban, academic institution, which has an annual patient volume of over 90,000 patients, requested more feedback regarding their productivity directly from residency leadership and through the annual Accreditation Council for Graduate Medical Education (ACGME) resident survey.^11^ In response, we developed an automated resident productivity dashboard, which has been distributed daily via email to the entire residency since 2016. The dashboard mimics the one provided to attending physicians by our department’s administrative group and includes a table of the following productivity data attributed to individual residents by name, as extracted from the electronic health record (EHR) every 24 hours: total number of patients seen; number of patients admitted; median time to admission order for admitted patients; and median time to discharge instruction printing for discharged patients. The dashboard displays this data for each specific shift, attributed to the individual resident working on a given day, and as such is dependent on fluctuations in variables such as patient volumes, case complexity, and bed availability. It is sent as a daily email to the departmental listserv, allowing a public, side-by-side comparison of individuals. There is no immediate functionality to generate a longitudinal report for oneself via the email (although this can be obtained through the software by an administrator), and dashboard data has not been used in the formal assessment of resident performance.

Objectives of our study were the following: 1) to assess residents’ perceptions of the productivity dashboard; and 2) to measure the impact of reviewing aggregate dashboard data on residents’ assessment of their own productivity.

## METHODS

We sent an anonymous electronic survey focusing on resident experience with the daily dashboard to post-graduate year (PGY) 1–3 residents in our four-year EM residency during an in-person, residency-wide retreat in July 2019 (Supplement 1). The survey was developed by residency leaders through an iterative process, which included final editing after piloting by residency members exempt from the study who provided feedback on survey questions. The PGY-4 residents were excluded as their supervisory role was too variable within the clinical structure of our residency (eg, PGY-4 residents may or may not electronically sign up for patients if they are supervising a junior resident).

The 10-question survey queried residents’ perceptions and perceived educational benefit of the daily dashboard, how often it was reviewed, how reflective it was of their actual performance, and how each resident felt their own productivity compared to that of their peers. After completing the first part of the survey, each resident was provided with their personal aggregate productivity data averaged over all shifts during the previous 10 months along with aggregated, matched peer-comparison data in a similar format to the daily dashboard, but with longitudinal data points rather than on a per shift basis. Residents were then asked again how they compared to their peers. Finally, residents were asked to identify additional quality and performance metrics that they would be interested in receiving. Most responses were collected on a five-point Likert scale, along with an option for write-in suggestions for improving the dashboard. This study was deemed exempt by the Yale University Institutional Review Board. All participants provided informed consent prior to beginning the survey.

We calculated summary statistics for the general survey questions. The distributions of pre- and post-comparison self-ratings were analyzed using the Wilcoxon signed-rank test with the Pratt modification for observed differences of zero.^12^ Free-text responses were not comprehensive enough to warrant formal qualitative analysis.

## RESULTS

**Figure 1:**
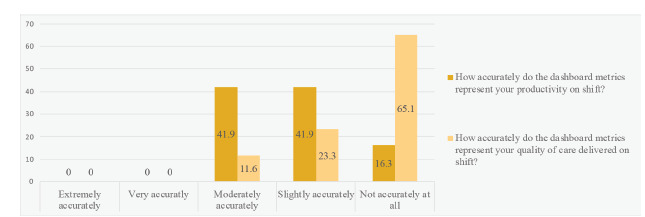
Residents’ perceptions of the accuracy of a productivity dashboard.

All 43 eligible PGY 1–3 residents completed the survey, for a response rate of 100%. One resident was ineligible due to participation as an investigator in the study. Thirteen (30%) residents reported “rarely” or “never” reviewing the dashboard. None felt the dashboard measured their productivity or quality of care “extremely accurately” or “very accurately” (Figure 1). Almost all residents expressed interest in receiving personalized lists of 72-hour returns (37, 86%) or in-hospital escalations of care within 24 hours (39, 91%).

Seven (16%) residents felt “very” or “extremely pressured” to change their practice patterns based on the metrics provided, while most felt moderate (15, 34.9%), slight (11, 25.6%), or no pressure at all (10, 23.3%). Twenty-eight (65%) would have preferred private feedback, rather than the public distribution of data. Most residents (18, 41.9%) felt neutral about how “helpful” the peer-comparison data provided during the survey was. Fifteen residents overall (35%), and 38% of residents reporting “rarely” or “never” looking at the dashboard, changed their self-perceived rank after viewing peer-comparison data. The overall change in how residents perceived themselves after review of the comparison data—ie, viewing themselves more positively or more negatively than before—did not show a significant trend in one particular direction (z = 0.71, *P* = 0.48).

Free-text feedback collected consisted of only five brief comments, including concerns about “gaming the system” resulting in inaccurate data collection on the dashboard and the department valuing “throughput over high quality, thorough care.”

## DISCUSSION

The development and dissemination of productivity data has been requested by our residents both informally and formally through the annual ACGME survey, and this is an area of interest to many residents and educators.^10^,^13^–^15^ The resident sentiment regarding our implementation, however, was mixed. Residents seemed skeptical of how accurately the data provided reflected their work, feeling it was less accurate in reflecting their quality of care than their productivity. The origin of this sentiment warrants further investigation since higher resident confidence in the fidelity of the data presented could drive higher future resident engagement with our dashboard.

Residents in a prior study appeared to have a more positive reception of their productivity metrics.^6^ One possible reason for this difference is that their data was provided privately, whereas ours made public the information to and about a potentially vulnerable population of trainees. Discomfort with comparison itself, however, does not make it unimportant or invalid, as most attending physicians will encounter at least some metric comparisons to a benchmark in their careers, and this may even be an intrinsic motivator for improvement.^1^

After comparing themselves to the mean productivity of their peers, about a third of residents revised their impressions, with fewer classifying themselves as average. While previously published findings have found that residents tend to overestimate their abilities,^16^–^18^ respondents in our study were equally likely to be optimistic or pessimistic about themselves: some shifted to a higher perceived productivity, while a slightly greater number shifted to a lower perceived productivity, although there was no significant trend in either direction. One possible explanation for the lack of significant change is that residents may already have had an overall accurate impression of themselves or because they did not trust the data provided and so did not update their impressions. Furthermore, those residents who “rarely” or “never” looked at the daily dashboard changed their self-rating after seeing the aggregate data 38% of the time, compared to 35% for the overall group. This raises the question of what impact looking at the dashboard more or less frequently may have on self-perceived productivity.

We did not compare actual productivity metrics before and after the review of aggregate peer-comparison data due to the survey’s anonymization procedure. Further studies could evaluate whether there is any correlation between resident self-perception and actual metrics, as well as whether review of the aggregate data had any effect on metrics after such an intervention.

We applied the insights derived from the survey to develop a revised resident dashboard, which is personalized and confidential to each resident and displays the resident’s metrics with anonymized peer- comparison data. It also contains follow-up lists of each resident’s patients who “bounce back” after discharge or have an escalation of care after admission. These lists link the resident directly to the patient’s chart in our facility’s EHR. The revised dashboard is currently undergoing pre-release testing.

## LIMITATIONS

As previously discussed, we did not include the PGY-4 class in our survey due to their supervisory role in our emergency department. In the future, to include input from the class closest to entering the workforce, leadership could standardize how and when supervising PGY-4 residents sign up for patients electronically.

Additionally, given that residents have had access to the daily dashboard showing data for individual shifts over the past three years, this survey was not the first time that they received their productivity data. However, it was the first time that they saw it in such aggregate format that is presumably less dependent on daily fluctuations in departmental factors. Certainly, the prior exposure may have already affected some residents’ perceptions of themselves. Receiving the daily dashboard may have a more significant effect; however, this was not within the scope of this study.

## CONCLUSION

Responding residents do not view patient-per-shift and patient-per-hour metrics as reflective of their true productivity or quality of care. Viewing the dashboard did not lead to any statistically significant changes in self-perceived resident productivity. This data highlights the need for expanding the resident conversation and education on metrics, given their frequent inclusion in attending workforce payment and incentive models. This exploration of resident perceptions of a metrics dashboard can be of use when designing similar dashboards for other institutions.

This work was presented as a poster at the Connecticut College of Emergency Physicians Scientific Assembly & Annual Meeting in September 2019.

## Supplementary Information
