# Supplementary material for: Resident Perceptions of a Publicly Disclosed Daily Productivity Dashboard
Source: West J Emerg Med. 2022 Jan 3;23(1):86–9. doi: 10.5811/westjem.2021.10.53874 (PMC8782132; doi:10.5811/westjem.2021.10.53874)
Supplement: Supplementary file 1 [file wjem-23-86-s001.docx]

Supplement 1.

ED Resident Sentiments on Learning Value of ED Daily Operational Dashboard Data

**Informed Consent** This is a survey study that will evaluate the perceived learning value of providing EM residents with their daily operational dashboard data. Your answers will be confidential and will not be used to evaluate your clinical performance. The survey is optional. It should take about 5 minutes to complete.   If you agree to participate in this survey, indicate your consent below.  If you do not agree to participate, close this survey now.

- **Yes, I agree to participate**

**When answering the following questions, think about the ED Resident Daily Dashboard that is currently emailed to you on a daily basis.**

Q1 **How often do you review the daily dashboard?**

- Every day
- A few times per week
- A few times per month
- Rarely
- Never

Q2 **My perceived performance compared to my peers is:**

- Much better than average
- Better than average
- About average
- Somewhat worse than average
- Much worse than average

Q3 **How accurately do you feel the dashboard metrics represent your productivity on a shift?**

- Extremely accurately
- Very accurately
- Moderately accurately
- Slightly accurately
- Not accurately at all

Q4 **How accurately do you feel the dashboard metrics represent the quality of care you deliver on a shift?**

- Extremely accurately
- Very accurately
- Moderately accurately
- Slightly accurately
- Not accurately at all

Q5 **How pressured do you feel to change your practice (e.g. try to work faster, etc.) based on the dashboard metrics?**

- Extremely pressured
- Very pressured
- Moderately pressured
- Slightly pressured
- Not pressured at all

Q6 **Would you rather receive data that is private (though perhaps less frequent)?**

- Yes
- No

**STOP!** Review your individual data before going on to the rest of the survey.

**When answering the following questions, think about the personalized data you just received.**

Q7 My performance compared to my peers was:

- Much better than average
- Better than average
- About average
- Somewhat worse than average
- Much worse than average

Q8 Rate your agreement with the following statement:
I found comparison data with my peers helpful.

- Strongly agree
- Somewhat agree
- Neither agree nor disagree
- Somewhat disagree
- Strongly disagree

Q9 I would be interested in receiving (check all that apply):

- Lists of my patients who bounce back to the ED within a certain time period such as 3, 7, or 21 days
- Lists of my patients who have an escalation of care or a rapid response within 24 hours of admission
- Lists of my patients who are made CMO or die during their admission to the hospital
- Lists of my patients who have contact with the follow-up nurse
- Other data (please comment below):

Q10 Other feedback you have for improving the feedback data you receive:

________________________________________________________________

________________________________________________________________

________________________________________________________________

________________________________________________________________

________________________________________________________________
